# Occult proximal femoral fracture with radiating leg pain masquerading as sciatica: a case report

**DOI:** 10.1186/s13256-023-03951-9

**Published:** 2023-05-25

**Authors:** Ji-yeon Lee, Akihito Oya, Osahiko Tsuji, Taro Umezu, Arihiko Kanaji, Yasuo Niki, Masaya Nakamura, Morio Matsumoto

**Affiliations:** Department of Orthopaedic Surgery, Keio University School of Medicine, 35 Shinanomachi,, Shinjuku-Ku, Tokyo 160-8582 Japan

**Keywords:** Occult fracture, Femur neck, Early diagnosis, Sciatica

## Abstract

**Background:**

Occult proximal femoral fractures do not appear as fracture lines in radiographs, causing misdiagnosis and delayed diagnosis unless additional imaging studies, such as computed tomography or magnetic resonance imaging, are performed. Here, we present a 51-year-old male with an occult proximal femoral fracture who experienced radiating unilateral leg pain that took 3 months to be diagnosed because his symptoms mimicked lumbar spine disease.

**Case presentation:**

A 51-year-old Japanese male experienced persistent lower back and left thigh pain after falling off a bicycle, and was referred to our hospital 3 months thereafter. Whole-spine computed tomography and magnetic resonance imaging revealed minute ossification of the ligamentum flavum at T5/6 without spinal nerve compression, but this did not explain his leg pain. Additional magnetic resonance imaging of the hip joint revealed a fresh left proximal femoral fracture without displacement. He underwent surgery for *in situ* fixation using a compression hip screw. Post-surgical pain relief was immediate.

**Conclusions:**

Misdiagnosis of occult femoral fractures as lumbar spinal disease may occur if distally radiating referred pain is present. Hip joint disease should be considered as a differential diagnosis in cases of sciatica-like pain with an unknown spinal origin and no specific findings on spinal computed tomography or magnetic resonance imaging accounting for the leg pain, especially following trauma.

## Background

Occult proximal femur fractures (OPFFs) exhibit normal radiographic findings and often occur in the older population because of bone fragility. Plain radiography should be primarily considered upon examining cases suspicious of this type of fracture with a history of trauma. However, because an occult fracture shows negative findings on a plain radiograph, a correct diagnosis may be difficult when accompanied by nonspecific symptoms, such as referred lower limb pain [1]. Here, we report the case of a left OPFF with a 3-month history of persisting lower limb pain after a trauma incident that was successfully surgically treated.

## Case presentation

### History and examination

A 51-year-old Japanese male experienced lower back and left thigh pain after falling off a bicycle. The first-contact doctor did not detect any obvious fracture line on a plain radiograph and followed up conservatively. However, the patient’s pain still persisted 1 month after the injury and without relief, so he visited a different nearby orthopedic clinic. Because the left thigh and lower extremity pain closely resembled sciatica originating from a lumbar spinal lesion, the orthopedic doctor performed lumbosacral magnetic resonance imaging (MRI). However, no significant lumbar spinal stenosis or herniated disc was observed that could account for his leg pain, so additional cervical and thoracic MRI and whole-spine computed tomography (CT) were conducted to screen for covert spinal lesions. The thoracic MRI revealed an ossified lesion of the ligamentum flavum at the T5/6 level, but it was too minute to compress a spinal nerve or induce any neuropathic pain. Furthermore, the whole-spine MRI findings could not account for his radicular pain. His pain persisted and gradually worsened. Because no diagnosis was able to be made, 3 months after his initial injury, he was referred to our hospital for a thorough and detailed examination.

On his initial visit to our hospital, the patient complained of lower back pain and radiating leg pain from the left buttock to the posterior thigh area. His left leg pain was also spreading to the lateral side of the lower leg and the dorsum of the foot. Physical examination indicated a positive straight leg raising test on the left side, with severe pain occurring at 20 degrees, and the Patrick test was also positive on the left side. There was no motor weakness or reflex abnormality. Notably, even with severe pain, he was an independent ambulator despite claudication.

**Fig. 1:**
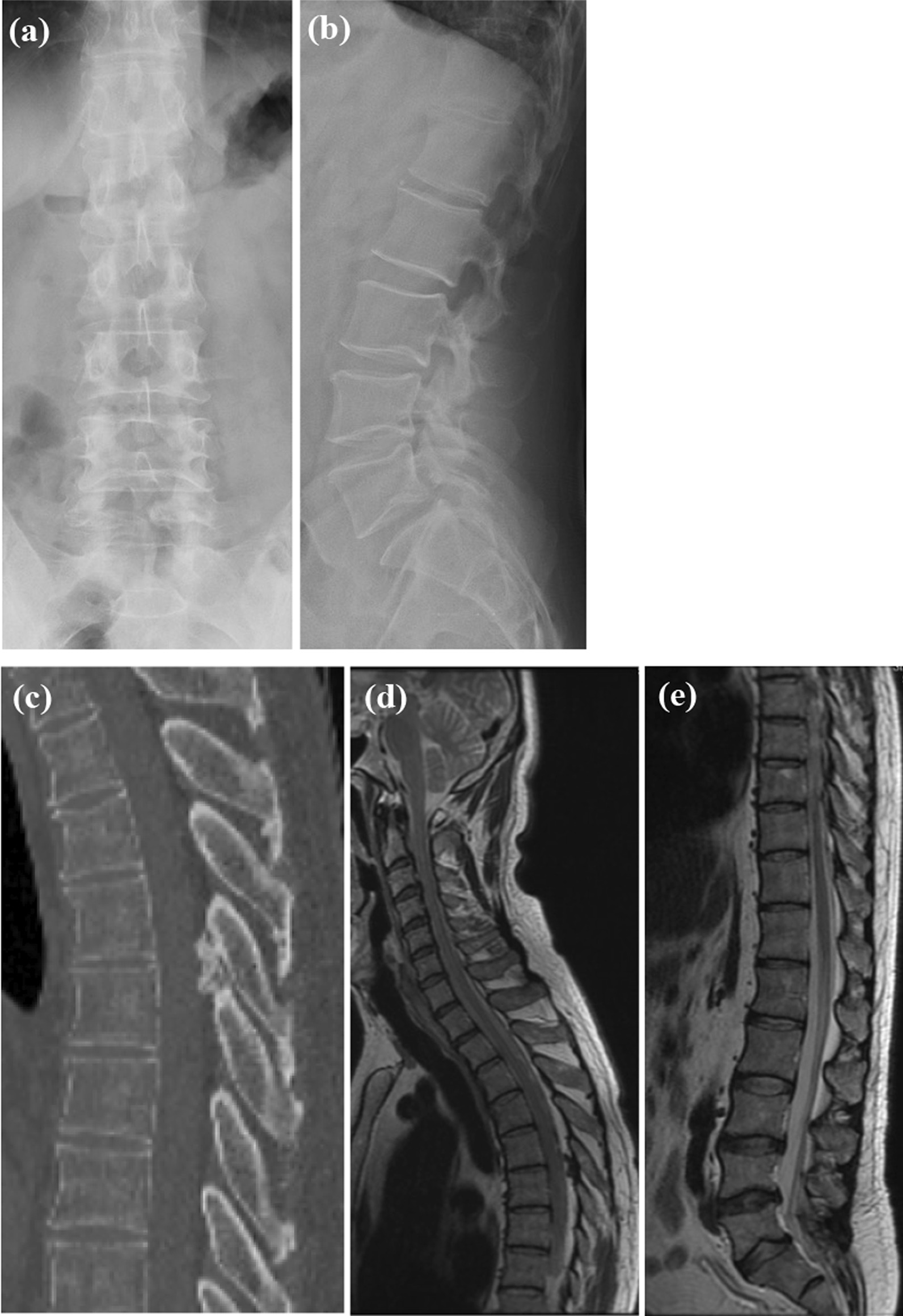
Imaging results of the injury. Anteroposterior view (**a**) and lateral view (**b**) of lumbar plain radiography performed after the injury. Sagittal view (**c**) of the thoracic spine CT and T2-weighted MRI of the cervical spine (**d**) and lumbar spine (**e**) 1 month after the injury. Degenerative changes in the lumbar spine and ossification of the ligamentum flavum at T5/6 were noted. However, there were no signs of significant spinal cord compression

**Fig. 2:**
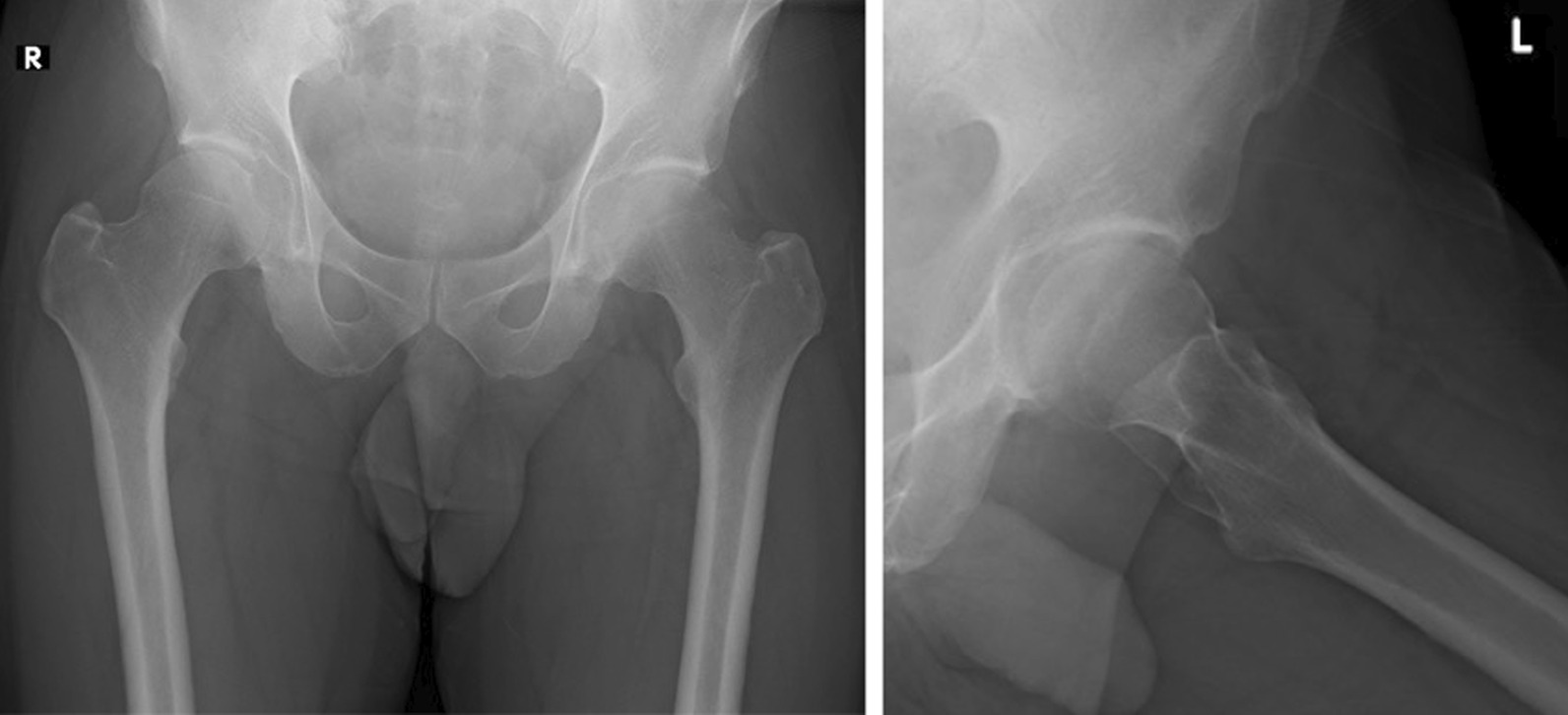
Plain radiography images taken 3 months after the injury. Anteroposterior view (left) and Lauenstein view (right) of the hip joint did not show any obvious abnormal findings

**Fig. 3:**
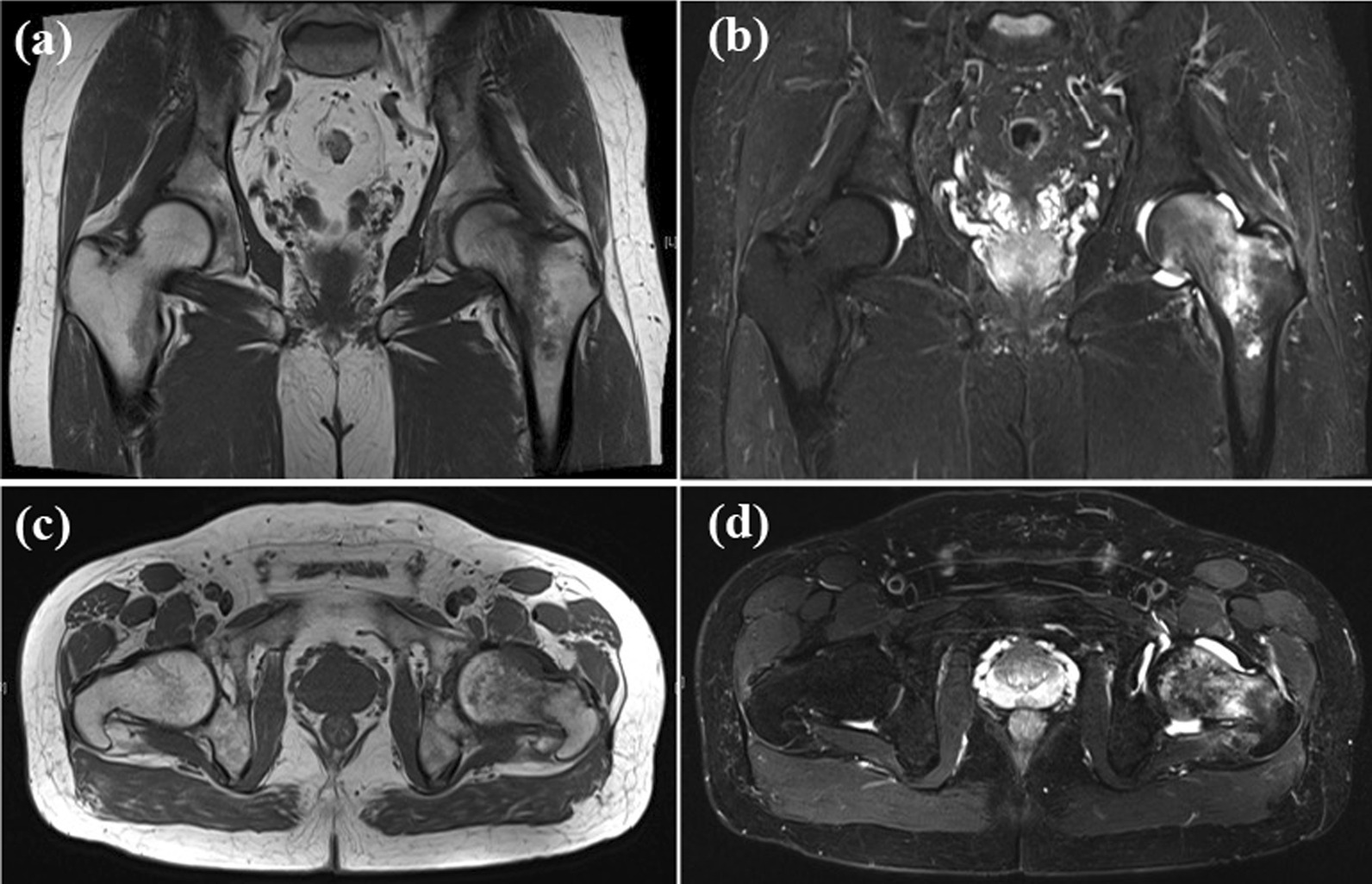
Images taken 3 months after the injury. Coronal view (**a**) and axial view (**c**) of hip joint T1-weighted MRI, and coronal view (**b**) and axial view (**d**) of hip joint short tau inversion recovery MRI. A fracture line with bone marrow edema was found in the left proximal femur

Reassessment of the serial spine and hip images showed degenerative changes in the lumbar spine and confirmed the absence of an identifiable fracture. The minute ossified lesion of the ligamentum flavum at T5/6 was identified on the CT and MRI images, but there were no compressive changes to the spinal canal (Fig. 1). We then performed hip radiographs at our hospital, but no significant findings, including fractures, were revealed (Fig. 2). Next, on the basis of his history of trauma without any spinal lesion, we performed hip joint MRI screening for hip and pelvic lesions. A fracture line with bone marrow edema at the left proximal femur exhibiting low T1 and high T2 signal intensity on the T2-weighted image (Fig. 3) was revealed, leading to the diagnosis of OPFF.

### Surgery and post-treatment course

**Fig. 4:**
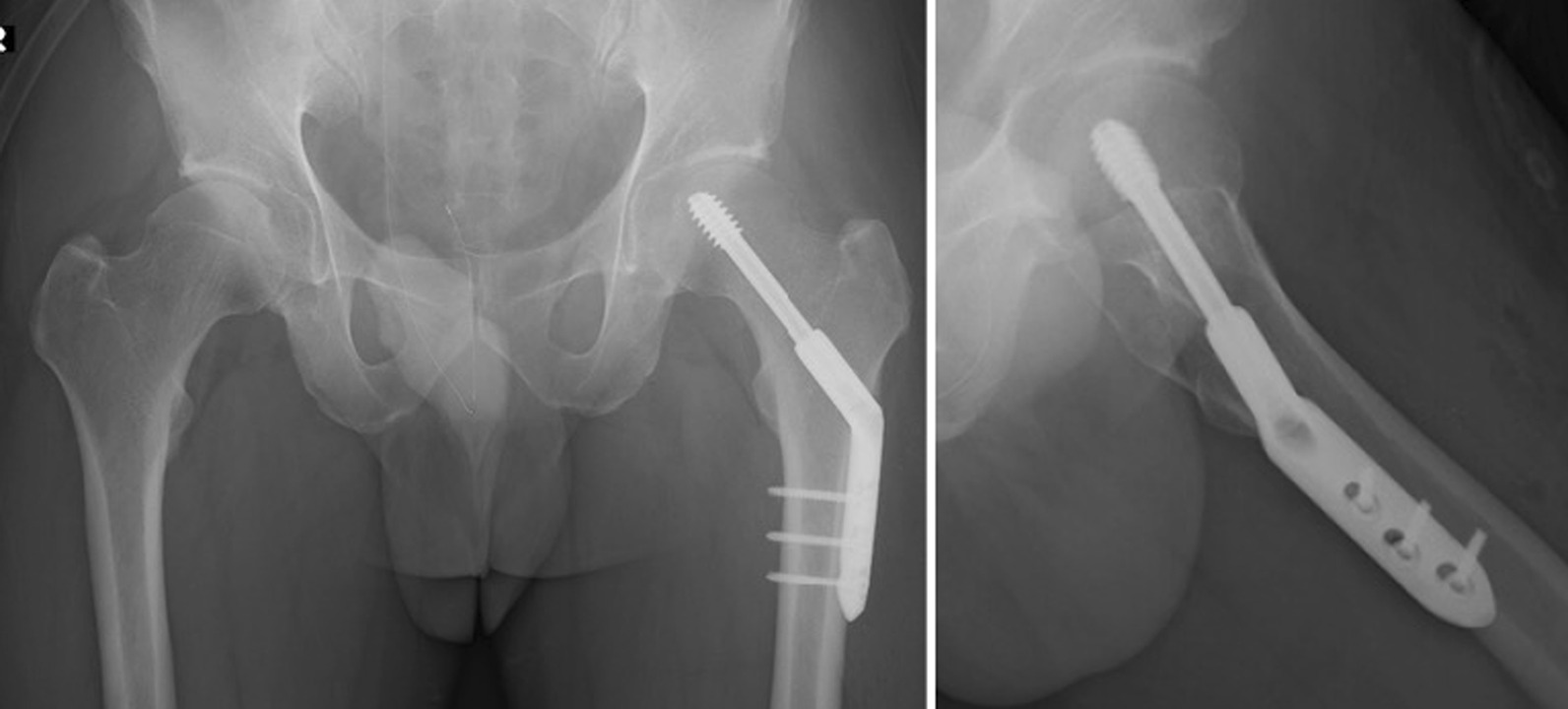
Postoperative images. Anteroposterior view (left) and Lauenstein view (right) of hip joint plain radiography showing that the compression hip screw was correctly inserted

**Fig. 5:**
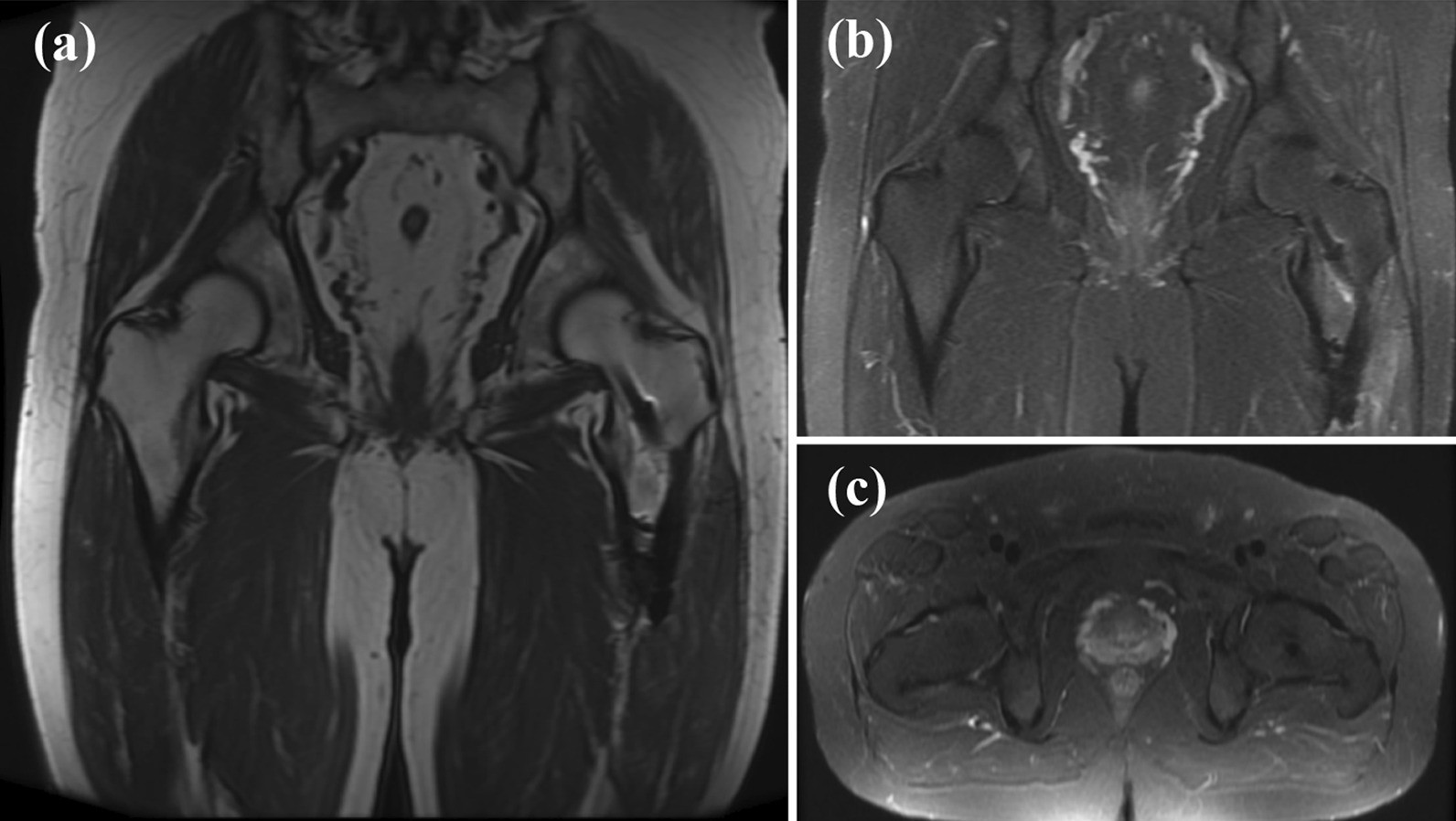
MRI performed 7 months after surgery. Coronal view (**a**) of hip joint T1-weighted MRI. Coronal view (**b**) and axial view (**c**) of hip joint short tau inversion recovery MRI. The fracture line in the proximal femur had disappeared

Because the patient’s pain had persisted for 3 months and gradually worsened, he underwent surgery under general anesthesia to stabilize the OPFF by osteosynthesis. We inserted a compression hip screw and fixed it *in situ* (Fig. 4). During the operation, the left lower limb was pulled using a traction table, and the left hip joint was passively moved, and no fracture instability was noted. Full weight-bearing walking from the day after the operation was permitted. He started walking with a T-shaped cane 5 days after surgery and was discharged 17 days after surgery. One month after surgery, pain relief was complete, and his gait had returned to normal. A postoperative MRI at 7 months showed the disappearance of the fracture line and of the bone marrow edema in the left proximal femur (Fig. 5).

## Discussion and conclusions

The Japanese Orthopedic Association guidelines for femoral neck and trochanteric fractures recommended MRI as the additional secondary examination for patients suspected of proximal femur fracture in trauma cases where no fracture line is visible upon plain radiography [2]. Compared with MRI, CT has fewer contraindications and is more accessible in emergency situations. However, if there is no or a small displacement, CT images may not always show the fracture line. There are several reports of misdiagnosed OPFF by CT findings that could only be diagnosed by MRI [2–5]. MRI is useful not only in diagnosing OPFF, but also in identifying the cause of hip joint pain. Oka *et al*. performed MRI on patients who complained of hip joint pain with unknown origin but did not show obvious abnormal findings on plain radiography, revealing fractures, such as those of the femoral neck, trochanter, and scrotum as well as soft tissue damage [6].

Early diagnosis and treatment of OPFFs are important because delayed diagnosis could affect the postoperative prognosis, especially in the older population. According to Novack *et al*. [7], if more than 4 days elapse between the injury and surgery, there is an increase in the postoperative hospital stay, in-hospital mortality rate, and the 1-month and 1-year mortality rate, respectively. Moreover, persistent severe chronic pain due to an occult fracture may result in central sensitization of the pain [8]. Thus, early diagnosis is clinically relevant.

Several factors, such as the patient’s medical history and nonspecific symptoms, can delay OPFF diagnosis. Hossain *et al*. reported that patients who live independently in their own homes are more likely to be correctly diagnosed compared with patients who require institutional care or have dementia [9].

Patients with hip joint disorders often complain of groin, buttock, and thigh pain [10]. Furthermore, pain from the buttock to the posterior side of the lower leg is a typical sign of spinal disease. However, Khan *et al*. reported that hip joint disorder could also cause pain in the anterior and posterior sides of the knees or lower legs [10]. Chronic hip joint pain can cause lumbar or lower leg pain that is similar to the referred pain of a lumbar lesion. Nakamura *et al*. reported that 55% of patients with osteoarthritis secondary to developmental dysplasia of the hip experienced referred pain in the thigh, knee, lower leg, or lower back. Additionally, 77% of patients with idiopathic osteonecrosis of the femoral head experienced referred pain [11, 12].

Our patient complained of pain not only in the left buttock and thigh, but also in the posterior side of the lower leg. Thus, sciatica with spinal disease was suspected at the first hospital visit. This delayed the diagnosis of OPFF because physicians first performed plain radiography and MRI of the spine.

We experienced a case of OPFF with difficulty in diagnosis. Even in patients showing typical spine-disease-like pain, neurologic findings must be checked for their appropriateness to the spinal lesion identified in the image. If inappropriate, a hip joint MRI should also be performed to screen for an occult femur fracture, as in this case.

## Data Availability

All data generated or analyzed during this study are included in this published article.

## Abbreviations

OPFF: Occult proximal femur fracture
MRI: Magnetic resonance imaging
CT: Computed tomography
